# Ergot in Diseased Conditions of the Prostate

**Published:** 1901-03

**Authors:** 


### Ergot in Diseased Conditions of the Prostate.

E. R. Corson, in *Merck's Archives,* recommends the following, which he finds useful, especially in congestion and inflammation caused by gonorrhea:

℞ Tinct. aconiti ........................ ʒi

Tinct. gelsemii ........................

Antipyrini ........................aa ℥ii

Ergotol. ........................ ℥i

Aquæ destil. q. s. ad........................ ℥iv

M. Sig.: one dessertspoonful of water every two or three hours. He states that the action of ergot is aided by a combination with a potash salt, generally the bromid.—*Journal American Medical Association.*

